# 17β-Hydroxysteroid Dehydrogenase Type 2 Expression Is Induced by Androgen Signaling in Endometrial Cancer

**DOI:** 10.3390/ijms19041139

**Published:** 2018-04-10

**Authors:** Chiaki Hashimoto, Yasuhiro Miki, Sota Tanaka, Kiyoshi Takagi, Misaki Fue, Zhulanqiqige Doe, Bin Li, Nobuo Yaegashi, Takashi Suzuki, Kiyoshi Ito

**Affiliations:** 1 Department of Obstetrics and Gynecology, Tohoku University Graduate School of Medicine, Sendai 980-8575, Japan; chiaki@med.tohoku.ac.jp (C.H.); tanakasouta@hotmail.co.jp (S.T.); reallylibin@gmail.com (B.L.); nobuo.yaegashi@gmail.com (N.Y.); 2 Department of Disaster Obstetrics and Gynecology, International Research Institute of Disaster Science (IRIDeS), Tohoku University, Sendai 980-8577, Japan; miki@irides.tohoku.ac.jp (Y.M.); fue@med.tohoku.ac.jp (M.F.); zhulanqiqige@med.tohoku.ac.jp (Z.D.); 3 Department of Pathology and Histotechnology, Tohoku University Graduate School of Medicine, Sendai 980-8575, Japan; k-takagi@med.tohoku.ac.jp (K.T.); t-suzuki@patholo2.med.tohoku.ac.jp (T.S.)

**Keywords:** 17β-hydroxysteroid dehydrogenase type 2, androgen, endometrial cancer, endometrioid endometrial adenocarcinoma

## Abstract

Endometrial cancer is one of the most common female pelvic cancers and has been considered an androgen-related malignancy. Several studies have demonstrated the anti-cell proliferative effect of androgen on endometrial cancer cells; however, the mechanisms of the anti-cancer effect of androgen remain largely unclear. 17β-hydroxysteroid dehydrogenase type 2 (17β-HSD2), which catalyzes the conversion of E2 to E1, is known to be upregulated by androgen treatment in breast cancer cells. In this study, we therefore focused on the role of androgen on estrogen dependence in endometrial cancer. Dihydrotestosterone (DHT) was found to induce *17β-HSD2* mRNA and protein expression in HEC-1B endometrial cancer cells. DHT could also inhibit cell proliferation of HEC-1B when induced by estradiol treatment. In 19 endometrioid endometrial adenocarcinoma (EEA) tissues, intratumoral DHT concentration was measured by liquid chromatography/electrospray tandem mass spectrometry and was found to be significantly correlated with 17β-HSD2 immunohistochemical status. We further examined the correlations between 17β-HSD2 immunoreactivity and clinicopathological parameters in 53 EEA tissues. 17β-HSD2 status was inversely associated with the histological grade, clinical stage, and cell proliferation marker Ki-67, and positively correlated with progesterone receptor expression. 17β-HSD2 status tended to be positively associated with androgen receptor status. In 53 EEA cases, the 17β-HSD2-positive group tended to have better prognosis than that for the negative group with respect to progression-free survival and endometrial cancer-specific survival. These findings suggest that androgen suppresses the estrogen dependence of endometrial cancer through the induction of 17β-HSD2 in endometrial cancer.

## 1. Introduction

Endometrial carcinoma is one of the most common female pelvic malignancies in developed countries and continues to show an increased incidence. In the United States alone, 60,050 new cases and 10,470 deaths were reported for endometrial carcinoma in 2016 [1]. It is well known that sex-steroid hormones play pivotal roles in the development of hormone-dependent carcinomas, including prostate, breast, and endometrial cancers. In the case of endometrial carcinoma, estrogen is known to play an important role in carcinoma development and progression. As previously demonstrated, excessive and/or prolonged exposure to unopposed estrogens increases the risk of endometrial carcinoma, especially the endometrioid type, also known as Type I endometrial carcinoma [2].

Furthermore, 17β-hydroxysteroid dehydrogenase (17β-HSD) plays an important role in determining intratumoral estrogen concentrations in both breast and endometrial cancers. 17β-HSD type 2 (17β-HSD2) preferentially catalyzes the conversion of testosterone and estradiol (E2) to androstenedione and estrone (E1), respectively [3,4]. On the other hand, 17β-HSD type 1 (17β-HSD1) is responsible for the conversion of E1 to E2 [3,4]. In breast cancer, it is suggested that 17β-HSD1 plays an important role in the intratumoral estrogen concentration. However, in endometrial cancer 17β-HSD1 expression is either undetectable or very weak, while 17β-HSD2 is essential for the maintenance of intratumoral estrogen concentrations [5].

Recently, 17β-HSD2 expression was found to be closely associated with androgen signaling through binding to the androgen receptor (AR) in breast cancer cells [6,7]. Takagi et al. [6] reported that E2-mediated proliferation in an AR-positive breast cancer cell line, T-47D, was significantly inhibited by dihydrotestosterone (DHT). E2-mediated proliferation was also associated with increased 17β-HSD2 expression, which was induced by DHT in a dose-dependent fashion. AR expression is also found in a large majority of endometrial cancers and is associated with a good prognosis [8,9]. In addition, we reported that 5α-reductase type 1 immunoreactivity was positively associated with the intratumoral level of DHT and was an independent prognostic factor in endometrial cancer [8]. However, the mechanisms of the anti-cancer effect of androgen remain largely unclear. In the present study, we therefore first examined the effect of DHT treatment on 17β-HSD2 expression in endometrial cancer cells in vitro followed by an examination of the correlation between the expression of 17β-HSD2 immunoreactivity and intratumoral androgen concentrations in 19 endometrioid endometrial adenocarcinoma (EEA) tissues, as measured by liquid chromatography/electrospray tandem mass spectrometry (LC-MS/MS). Furthermore, the relationship between 17β-HSD2 expression and clinicopathological parameters, including patient outcomes, was evaluated in an additional 53 EEA patients.

## 2. Results 

**Figure 1:**
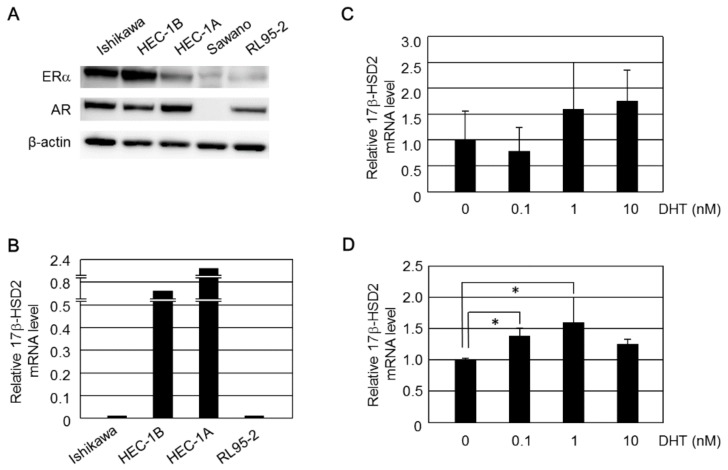
(**A**) Expression of ERα and AR proteins in endometrial cancer cell lines. ERα and AR proteins were detected by Western blotting. (**B**) Expression of *17β-HSD2* mRNA in endometrial cancer cell lines. *17β-HSD2* mRNA expression was examined using qRT-PCR. (**C**) Effect of DHT treatment on *17β-HSD2* mRNA expression in Ishikawa cells; (**D**) effect of DHT treatment on *17β-HSD2* mRNA expression in HEC-1A cells. ‘0’ denotes vehicle treatment alone; * *p* < 0.05.

Expression levels of ERα, AR, and 17β-HSD2 in endometrial cancer cell lines were examined. Expression of ERα and AR were detected by Western blotting (Figure 1A). Relatively high levels of ERα protein were detected in both Ishikawa and HEC-1B cells compared to those in HEC-1A, Sawano, and RL95-2 cells (Figure 1A). AR protein expression was detected in all cell lines except for Sawano (Figure 1A). Expression of *17β-HSD2* mRNA evaluated by qRT-PCR is shown in Figure 1B. *17β-HSD2* mRNA was markedly overexpressed in HEC-1A (Figure 1B). Relatively high expression levels of *17β-HSD2* mRNA were also detected in HEC-1B compared to Ishikawa and RL95-2 cells (Figure 1B). Based on these results, we employed Ishikawa, HEC-1A, and HEC-1B cells in subsequent *17β-HSD2* mRNA induction assays.

### 2.1. Induction of 17β-HSD2 Expression by DHT

*17β-HSD2* mRNA levels in Ishikawa cells were increased by more than 1.5-fold, compared to control cells, when treated with both 1 nM and 10 nM DHT; however, these differences were not statistically significant (Figure 1C). DHT (0.1 nM and 1 nM) treatment significantly increased *17β-HSD2* mRNA in HEC-1A (*p* < 0.05). However, estradiol did not stimulate cell proliferation in HEC-1A (data not shown). It is possible that the ERα was not functional because its expression level was low in HEC-1A cells when compared to Ishikawa and HEC-1B (Figure 1A).

**Figure 2:**
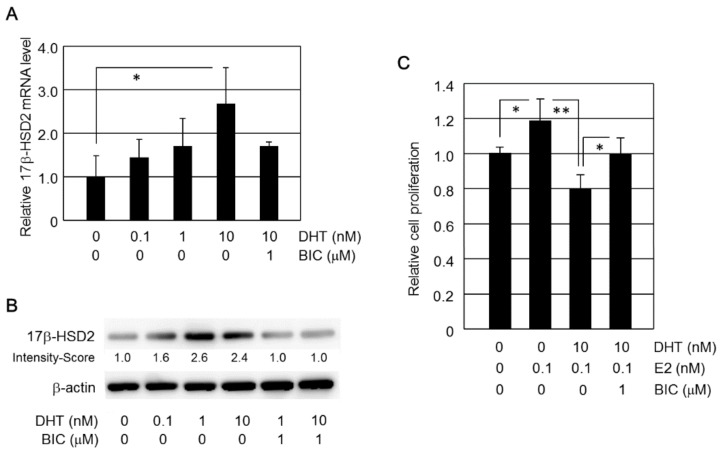
(**A**) Effect of DHT treatment on *17β-HSD2* mRNA expression in HEC-1B cells. *17β-HSD2* mRNA expression was examined using qRT-PCR. BIC, bicalutamide; ‘0’ denotes vehicle treatment alone; * *p* < 0.05. (**B**) Effect of DHT treatment on 17β-HSD2 protein expression in HEC-1B cells. 17β-HSD2 protein was detected by Western blotting. BIC, bicalutamide; ‘0’ denotes vehicle treatment alone. (**C**) Effect of DHT and E2 on HEC-1B cell proliferation. BIC, bicalutamide; ‘0’ denotes vehicle treatment alone; * *p* < 0.05; ** *p* < 0.01.

Expression levels of *17β-HSD2* mRNA in HEC-1B cells were increased by DHT in a dose-dependent manner, with significant differences, compared to the control, observed from 10 nM (*p* < 0.001) (Figure 2A). The effect of DHT treatment on *17β-HSD2* mRNA induction was significantly inhibited by co-treatment with 1 μM bicalutamide (*p* < 0.001, Figure 1A). Bicalutamide (1 μM) alone did not significantly change *17β-HSD2* mRNA levels in HEC-1B cells (data not shown). In Western blot analysis, relatively high levels of 17β-HSD2 immunoreactivity were detected in HEC-1B cells treated with 1 nM and 10 nM DHT. The induction of 17β-HSD2 immunoreactivity was inhibited by 1 μM bicalutamide treatment (Figure 2B).

### 2.2. DHT Inhibits E2-Induced Cell Proliferation in HEC-1B Cells

HEC-1B cells were treated with or without E2 (100 pM), DHT (10 nM), and bicalutamide (1 μM) for 4 days (Figure 2C). The relative cell proliferation was evaluated as a ratio (%) compared with control cells. HEC-1B cell proliferation was significantly higher in E2-treated (0.1 nM) HEC-1B cells than in control HEC-1B cells. DHT (10 nM) treatment significantly inhibited the increase in HEC-1B cell proliferation induced by E2 (0.1 nM). This DHT inhibitory effect on E2-induced cell proliferation was significantly prevented by co-treatment with bicalutamide (1 μM).

### 2.3. Immunohistochemistry of 17β-HSD2 and AR in EEA

**Figure 3:**
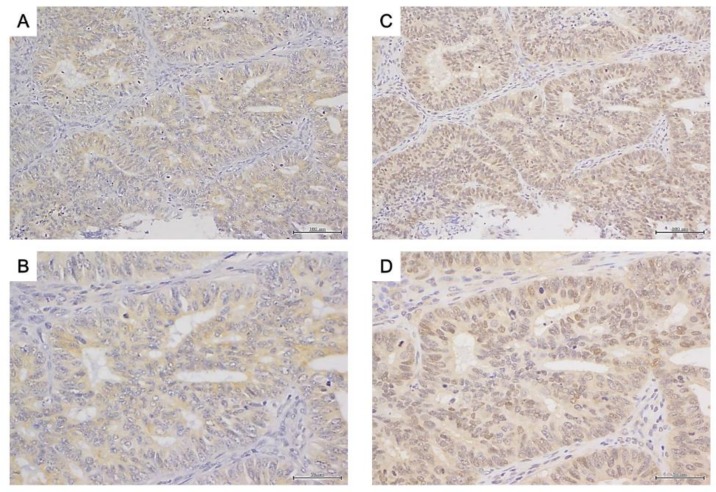
Immunohistochemistry for 17β-HSD2 (endometrioid endometrial adenocarcinoma: grade 1, stage IA) ((**A**) low magnification; (**B**) high magnification) and AR (endometrioid endometrial adenocarcinoma: grade 1, stage IA) ((**C**) low magnification; (**D**) high magnification). Scale bar, 100 μm (**A**,**C**) and 500 μm (**B**,**D**).

17β-HSD2 immunoreactivity was detected in the cytoplasm of carcinoma cells (Figure 3A), while AR immunoreactivity was detected in the nuclei of carcinoma cells (Figure 3B). In the 19 cohort 1 EEA tissues, staining for 17β-HSD2 was positive in 7 cases (36.8%). All 19 samples were AR positive. In the 53 cohort 2 EEA tissues, staining for 17β-HSD2 was positive in 19 cases (35.8%) (Table 1). Staining for AR was positive in 40 cases (75.5%) (Table 1).

### 2.4. 17β-HSD2 Expression Is Correlated with DHT Concentration in Cancer Tissues

**Figure 4:**
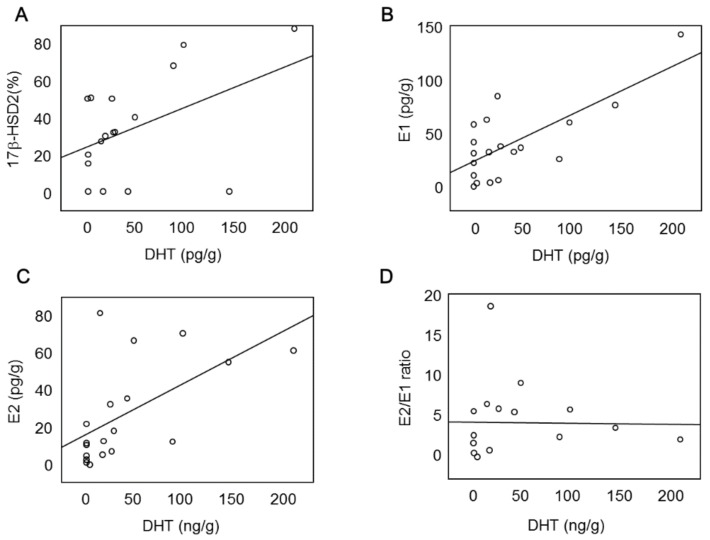
(**A**) Correlation between 17β-HSD2 (%) and intratumoral DHT concentration (pg/g) in 19 endometrioid endometrial adenocarcinoma cases. (**B**–**D**) Correlation between intratumoral DHT and E1 (**B**), E2 (**C**) concentrations (pg/g) or E2/E1 ratio (**D**) in 19 endometrioid endometrial adenocarcinoma cases.

We evaluated the correlation between the areas of immunoreactivity of 17β-HSD2 and intratumoral DHT concentration in 19 EEA tissues (Figure 4). As shown in Figure 4A, the area of immunoreactivity of 17β-HSD2 (%) was positively correlated with the intratumoral DHT concentration in 19 EEA tissues. We also examined the intratumoral concentration of E2 and E1 in 19 EEA tissues. As shown in Figure 4B,C, the intratumoral E2 concentration did not correlate with DHT concentration. Meanwhile, the intratumoral E1 concentration was significantly positively correlated with the intratumoral DHT concentration. However, E2/E1 ratio was not correlated with the intratumoral DHT concentration (Figure 4D).

### 2.5. 17β-HSD2 Clinicopathological Parameters in EEA

The correlations between 17β-HSD2 immunoreactivity and clinicopathological parameters are summarized in Table 1. Nineteen of the 53 cohort 2 EEA tissues (35.8%) were 17β-HSD2-positive. The 17β-HSD2 status was inversely associated with the histological grade (*p* < 0.05), clinical stage (stage I and II vs. stages III and IV), and the Ki-67 LI (*p* < 0.05), and positively correlated with the PR LI (*p* < 0.01). 17β-HSD2 status tended to be positively associated with AR status (*p* = 0.0629).

**Figure 5:**
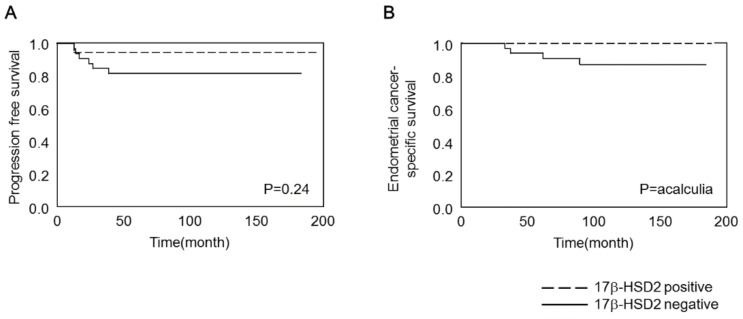
Correlation between 17β-HSD2 status and progression-free survival (**A**) or endometrial cancer-specific survival (**B**) for 53 patients with endometrioid endometrial adenocarcinoma. 17β-HSD2 status was evaluated via immunohistochemistry, and immunoreactivity greater than 50% was considered positive. Dates were compared statistically using the log-rank test. *p* < 0.05 was considered significant. The median value of 17β-HSD2 score was as follows: total, 40 ± 20.30 (range 0–70%); positive group, 50 ± 6.97 (range 50–70%); negative group, 20 ± 14.45 (range 0–40%).

The progression-free survival (PFS) and endometrial cancer-specific survival (ECSS) curves of the patients were constructed using the Kaplan–Meier method (Figure 5). In this analysis, we examined PFS rather than disease-free survival to include stage IVB lesions, for which distant metastases remained after the primary surgery. We also examined ECSS rather than the overall survival because one patient death was unrelated to endometrial cancer and occurred in the absence of relapse. Patients positive for 17β-HSD2 immunoreactivity tended to have better prognosis than those negative for 17β-HSD2 immunoreactivity, with respect to PFS (Figure 5A) and ECSS (Figure 5B).

## 3. Discussion

To the best of our knowledge, this is the first report that 17β-HSD2 was induced by DHT in endometrial cancer. It has long been known that *17β-HSD2* mRNA expression is induced by DHT treatment in prostate (LNCaP) [10] and breast (T-47D) [6] cancer cell lines. In invasive lobular carcinoma (ILC) of the breast, significant positive association was reported between the status of androgenic enzymes, which are 17β-HSD type 5 (17β-HSD5) and 5α-reductase, and 17β-HSD2 [7]. In addition, 17β-HSD2 expression was inversely correlated with tumor size of breast ILC [7]. These findings, combined with the results from our present study, indicate that DHT acted, at least in part, through 17β-HSD2 induction and decreasing intratumoral E2 concentrations, to influence anti-proliferative effects in breast and endometrial cancer. Takagi et al. [6] also reported that E2-mediated proliferation was significantly inhibited by DHT in AR-positive T-47D cells. In this instance, proliferation was also associated with an increase in 17β-HSD2 expression levels [6]. In addition, local E2 concentrations were inversely associated with 17β-HSD2 status in breast carcinoma tissues [6]. In our study, the cell proliferative effect of E2 was significantly inhibited by DHT treatment in HEC-1B cells. Therefore, it is considered that intratumoral estrogen concentrations were reduced by 17β-HSD2, which was induced by DHT treatment in both endometrial and breast cancers. In our study, DHT did not have a significant effect on 17β-HSD2 expression in Ishikawa cells, which had very low levels of 17β-HSD2 expression. We believe that stable results were not obtained because of very low levels of 17β-HSD2 expression in Ishikawa cells.

In the present study, an inverse correlation between intratumoral E2 and DHT was not observed in 19 EEA tissues examined owing to the small number of cases. Furthermore, both intratumoral E1 and E2 concentrations positively correlated with DHT concentration in all 19 EEA tissues. However, the E2/E1 ratio also did not correlate with DHT concentration for the 19 EEA cases. It is well-established that in breast cancer tissues, circulating androstenedione is converted by aromatase to E1 [11,12]. Intratumoral E1 is then further converted to E2 by 17β-HSD1 activity in breast cancer tissues [11,12]. Therefore, androstenedione has a key role in intratumoral estrogen synthesis in estrogen-dependent breast cancer. On the other hand, in endometrial cancer, E1 is not converted to E2 because of lack of 17β-HSD1 expression [13]. As expected, there was no 17β-HSD1 immunoreactivity in 19 EEA tissues examined in our study (data not shown). Therefore, testosterone to E2 conversion is an important pathway for intratumoral estrogen synthesis in endometrial cancer. It is also well-known that 17β-HSD2 converts testosterone into androstenedione, whereas 17β-HSD5 produces testosterone from androstenedione [14,15]. Therefore, these findings, in addition to the results from our study, suggest that 17β-HSD2 is a key player in intratumoral estrogen and androgen production. Further examination of the correlation between 17β-HSD2 and other estrogen/androgen-related enzymes is, however, required to clarify the role of 17β-HSD2 in the intratumoral hormone environment of endometrial cancer.

In breast cancer, 17β-HSD2 expression was reported to show significant association with decreased risk to develop late relapse [16,17]. We have reported that 17β-HSD2 immunoreactivity was detected in 37% of endometrial carcinoma cases and correlated with 17β-HSD2 enzymatic activity and expression of *17β-HSD2* mRNA [13,18]. In a normal endometrium, it was reported that both 17β-HSD2 protein and mRNA were present at all the stages of the secretory phase, but not in the endometrial mucosa during the proliferative phase [18,19]. Several studies consistently detected *17β-HSD2* mRNA expression in endometrial carcinoma compared to the adjacent normal endometrium at menopause [20,21,22]. Therefore, the 17β-HSD2 enzyme may play an important role in modifying the balance of estrogen production in endometrial carcinoma. In this study, 17β-HSD2 status was inversely associated with the histological grade, clinical stage (stage I and II vs. stages III and IV), and the Ki-67 LI (Table 1). The 17β-HSD2 positive cases had lower levels of Ki-67 LI; therefore, 17β-HSD2 possibly inhibits cell proliferation in EEA cells. The 17β-HSD2 positive group tended to have good prognoses, but a significant difference between them was not observed. In 17β-HSD2 positive cases, there were no deaths. Further studies, including a larger number of cases and longer periods of follow-up, are, therefore, warranted to elucidate the functional significance of 17β-HSD2 in endometrial cancer.

17β-HSD2 is considered as a DHT-induced gene in endometrial cancer cells, although androgen-responsive genes are not currently characterized in endometrial cancer cells. In the present study, DHT was found to induce 17β-HSD2 expression in HEC-1B cells. To the best of our knowledge, these findings have not been previously reported. Results obtained in our study suggest that the androgen signal plays a crucial role in the estrogen-dependency of ER-positive endometrial cancer cells. AR target genes such as *XBP1*, *MYC*, *ZBTB16*, and *UHRF1* were previously reported to be induced by DHT treatment in AR-positive endometrial cancer MFE-296 cells [23]. It was also reported that *XBP1* and *UHRF1* are related to tumor growth or carcinogenesis in endometrial cancer [24,25]. Furthermore, several studies demonstrated that tumor suppressor genes were directly induced by androgen treatment through AR genomic action in breast and prostate cancers [26,27]. It has also been suggested that androgen and its receptor signal may exert anti-tumor effects on endometrial cancer [14,15]. Further studies, such as comprehensive gene expression analysis, are, however, required to clarify the role that androgens play in the pathogenesis of endometrial cancer.

## 4. Materials and Methods

### 4.1. EEA Patient and Tissue Preparation

A total of 72 endometrial endometrioid adenocarcinoma tissues (EEA) of postmenopausal women were retrieved from the surgical pathology files of Tohoku University Hospital, Sendai, Japan. Both fresh-frozen and formalin-fixed paraffin-embedded (FFPE) specimens were available for 19 patients operated between 2012 and 2013 (cohort 1). The other 53 EEA tissues were available only as FFPE samples and were collected between 1993 and 2003 (cohort 2). The Ethics Committee of Tohoku University School of Medicine approved the research protocol of this examination (No. 2013-1-265). The clinicopathological parameters of the 19 patients in cohort 1 are summarized in Table S1. We measured E2, E1, and DHT concentration using LC-MS/MS in the 19 EEA fresh-frozen samples. The clinicopathological features of the 53 patients in cohort 2, for whom only FFPE tissues were available, are summarized in Table S2. None of the patients received radiation therapy, hormone therapy, or chemotherapy before surgery. All 72 EEA samples were post-menopausal to exclude the effect of serum hormonal concentration.

In Tohoku University Hospital, the standard primary treatment is surgery consisting of total abdominal hysterectomy, salpingo-oophorectomy, and pelvic and/or para-aortic lymphadenectomy with peritoneal washing cytology. Among 19 and 53 patients in cohort 1 and 2, 12 patients in cohort 1 and 42 patients in cohort 2 underwent lymphadenectomy, respectively. The lymph node metastasis was shown in four patients in cohort 1 and three patients in cohort 2, respectively. Pathological diagnosis including evaluation of pathological types and histologic grades was evaluated independently by pathologist and cytopathologist (S.T., K.I.). We diagnosed the patients according to the FIGO 2009 staging guidelines (FIGO Committee on Gynecologic Oncology, 2009).

Among 53 patients in cohort 2, 31 patients received pelvic radiation therapy (50 Gy) or 3–6 courses of chemotherapy [cisplatin-based combination regimen CAP (60–70 mg/m^2^ cisplatin, 40 mg/m^2^ doxorubicin, and 500 mg/body weight cyclophosphamide, triweekly)]. Among 19 patients in cohort 1, 10 patients received 3–6 courses of chemotherapy [AP (60 mg/m^2^ adriamycin and 50 mg/m^2^ cisplatin, triweekly) or TC (175 mg/m^2^ Taxol and area under the curve 5 carboplatin, triweekly)] after surgery. Patients diagnosed with early-stage and low-grade disease (stage IA, grade 1; or stage IA, grade 2) did not receive any adjuvant therapy. Patients with poor performance status also had no any adjuvant therapy. None of the patients in cohorts 1 and 2 received hormonal therapy after surgery.

### 4.2. Cell Lines and Chemicals

Ishikawa 3-H-12, HEC-1A, and HEC-1B were obtained from the Japanese Collection of Research Bioresources Cell Bank, National Institutes of Biomedical Innovation, Health and Nutrition (Osaka, Japan). RL95-2 (CRL-1671) was obtained from the American Type Culture Collection (Manassas, VA, USA). Sawano was obtained from the RIKEN BioResource Center (Tsukuba, Japan). Ishikawa 3-H-12, HEC-1A, and HEC-1B were cultured in MEM (Sigma-Aldrich, St. Louis, MO, USA) with 10% fetal bovine serum (FBS; Nichirei, Tokyo, Japan). HEC-1B cells were cultured in phenol red-free DMEM/F12 (Sigma-Aldrich) medium containing 10% dextran-coated charcoal (DCC)-FBS for three days before experimental treatment. DHT and E2 were purchased from Wako Pure Chemical Industries (Osaka, Japan). Bicalutamide, was purchased from Abcam (Cambridge, UK). Bicalutamide, which is well established as an androgen receptor antagonist, was employed in the range of 1 μM to 10 μM in several in vitro studies [28,29,30]. 10 μM bicalutamide had a cytotoxic effect at 24 h on HEC-1B cells (data not shown). Therefore, in this study, we used 1 μM bicalutamide to block androgen receptor signals. The status of cell proliferation was determined using the WST-8 (2-[2-methoxy-4-nitrophenyl]-3-[4-nitrophenyl]-5-[2,4-disulfophenyl]-2H-tetrazolium monosodium salt) method (Cell Counting Kit-8; Dojindo, Kumamoto, Japan).

In general, the E2 concentration used in in vitro studies ranged from 0.1 nM to 10 nM [31,32,33]. In this study, we used 0.1 nM E2, which was within the range of E2 levels found in EEA examined in this study. We used 0.1–10 nM DHT, the range used for in vitro experiments [6,29,30], for the *17β-HSD2* mRNA induction assay. A concentration of 10 nM DHT was approximately 10 times the average intratumoral concentration in EEA examined in this study. It is considered that 10 nM DHT did not exert a significant pharmacological effect in this examination due to the following reasons: DHT may be used at doses up to 100 nM [30]; the effect of DHT was inhibited by AR antagonist (Figure 2); and the induction of *17β-HSD2* mRNA was also obtained by 1 nM DHT treatment, though the difference was not statistically significant (Figure 2).

### 4.3. Real-Time PCR

Total RNA was extracted using TRIzol reagent (Molecular Research Center, Cincinnati, OH, USA), and cDNA synthesized using a QuantiTect reverse transcription kit (Qiagen, Hilden, Germany). Real-time PCR was carried out using the LightCycler System and FastStart DNA Master SYBR Green I (Roche Diagnostics, Mannheim, Germany). The PCR primer sequences for *17β-HSD2* and the *ribosomal protein L13A* (*RPL13A*) were as follows; *17β-HSD2* (NM_002153): forward 5′-CAAAGGGAGGCTGGTGAA-3′ and reverse 5′-TTGAGGACCTCTGTGTATTT-3′; and *RPL13A* (NM_012423): forward 5′-CCTGGAGGAGAAGAGGAAAGAGA-3′ and reverse 5′-TTGAGGACCTCTGTGTATTTGTCAA-3′. PCR products were purified and subjected to direct sequencing to verify amplification of the correct sequences. *17β-HSD2* mRNA levels were determined as a ratio of the reference *RPL13A* mRNA levels (%). We used only one housekeeping gene for normalization of quantitative PCR.

### 4.4. Western Blotting

Protein was extracted from cultured cells using Mammalian Protein Extraction Reagent (Pierce Biotechnology, Rockford, IL, USA) added with Halt Protease Inhibitor Cocktail (Pierce Biotechnology). The protein extracted from whole cell (20 μg) was analyzed by SDS-PAGE (10% acrylamide gel) and transferred onto Hybond P polyvinylidene difluoride membrane (GE Healthcare, Little Chalfont, UK). Primary antibodies employed in this study were anti-human 17β-HSD2 (10978-1-AP; Proteintech, Chicago, IL, USA) and anti-human β-actin (AC-15; Sigma-Aldrich). Antibody–protein complexes were detected using ECL plus Western Blotting Detection reagents (GE Healthcare), and visualized with LAS-1000 image analyzer (Fuji Photo Film, Tokyo, Japan). The intensity of each band of 17β-HSD2 was quantitated by using the Imaging Analyzer (Lumina Vision; MITANI CORPORATION, Tokyo, Japan). The data were demonstrated as an intensity score in Figure 2. 

### 4.5. Immunohistochemistry

Rabbit polyclonal antibody against 17β-HSD2 (10978-1-AP) was purchased from Proteintech. Mouse monoclonal antibodies against androgen receptor (AR; AR441), estrogen receptor (ER; ER1D5), progesterone receptor (PR; MAB429), and Ki-67 (MIB1) were purchased from DAKO (Carpinteria, CA, USA), Immunotech (Marseille, France), Chemicon (Temecula, CA, USA), and DAKO, respectively. A Histofine Kit (Nichirei), which employs the streptavidin–biotin amplification method, was used for immunohistochemistry. The antigen–antibody complex was visualized with 3,3′-diaminobenzidine (DAB) solution (1 mM DAB, 50 mM Tris–HCl, pH 7.6, and 0.006% H_2_O_2_) and counterstained with hematoxylin.

### 4.6. Evaluation of Immunoreactivity

The immune expression of AR, PR, ER, and Ki-67 was detected in the nuclei of endometrial cancer cells. We counted more than 500 carcinoma cells from at least three representative high-power fields (×400). The percentage of immunoreactive cells [labeling index (LI)] was evaluated. LI more than 10% was evaluated as being a positive case according to previous reports [34,35]. 17β-HSD2 immunoreactivity was detected in the cytoplasm of carcinoma cells, which were evaluated using a semi-quantitative score such as positive and negative according to previous reports [13,18]. Taking both intensity of immunoreactivity and expression areas into consideration, specimens stained with more than 50% immunoreactivity for 17β-HSD2 were evaluated as a positive case, and those with less than 50% immunoreactivity were determined as a negative case [13,18]. The immunohistochemistry of 17β-HSD2 was reported to be significantly associated with its enzyme activity and mRNA expression, when the cutoff value of the immunohistochemical score was 50% [18].

### 4.7. LC-MS/MS

The intratumoral concentrations of DHT, E2, and E1 in 19 endometrial specimens were measured using LC-MS/MS at ASKA Pharma Medical Co. Ltd. (Kawasaki, Japan), as described previously [6,36]. Approximately 1 cm^3^ specimens were obtained immediately after hysterectomy and cryopreserved at −80 °C in liquid nitrogen. Pathological diagnoses were conducted using an adjacent section of tissue. For all of these specimens we employed LC-MS/MS analysis, which confirmed that the proportion of carcinoma cells was at least 80%. The weights of tissue specimens used for LC-MS/MS analysis were 9–100 mg (mean ± standard deviation [SD]; 49 ± 21 mg). Frozen tissues were homogenized in 1 mL distilled water. Then, the steroid fraction derived from these samples was extracted by diethyl ether. LC system (Agilent 1100, Agilent Technologies, Waldbronn, Germany) coupled with an API 4000 triple stage quadrupole mass spectrometer (Applied Biosystems, Concord, ON, Canada) operated with electron spray ionization in the positive ion mode were employed in this analysis. Chromatographic separation was performed on a Cadenza CD-C^18^ column (3 × 150 mm, 3.5 mm, Imtakt, Kyoto, Japan). The detection limits of E2, E1, and DHT were 1.0. 2.0, 1.0 pg/g, respectively. Values less than the detection limit were assigned 0.0 pg/g.

### 4.8. Statistical Analyses

Statistical analyses were carried out using JMP Pro12 (SAS Institute Japan, Tokyo, Japan). The statistical significance of correlations between the 17β-HSD2 status and the DHT concentration in EEA were examined using the Wilcoxon test. The statistical significance of correlations between 17β-HSD2 status and other clinicopathological parameters [grade, stage, lymphovascular invasion (LVI), myometrial invasion (MI), AR status, ER status, PR status, and Ki-67 labeling index (LI)] were examined using the χ^2^ and Wilcoxon tests. Univariate analyses were examined using the Kaplan–Meier method and the log-rank test. *p*-values < 0.05 were considered statistically significant in this study.

## 5. Conclusions

**Figure 6:**
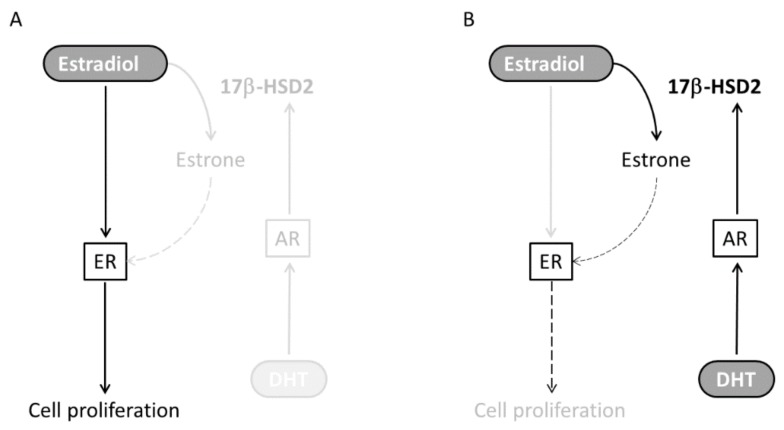
(**A**) In endometrial cancer cells, estradiol promotes cancer cell proliferation through binding to estrogen receptors (ER). (**B**) Androgen [dihydrotestosterone (DHT)] induces 17β-HSD2 expression through binding to androgen receptors (AR). When estradiol is converted into the weaker estrone, by 17β-HSD2, the cell proliferative activity of ER signal is diminished.

E2 is known as a major substrate for 17β-HSD2. 17β-HSD2 catalyzes the oxidation of the highly active E2 into the weak estrone, which has a very low affinity to ER compared to E2. In our study, HEC-1B cell proliferation was increased by E2 treatment. We found that induction of 17β-HSD2 occurred in HEC-1B cells when the cells were treated with DHT with E2, and E2 co-treated was converted into the weaker form, E1 (Figure 6). This finding suggests that DHT decreased E2-dependent endometrial cancer proliferation.

## Figures and Tables

**Table 1:** Correlation between 17β-HSD2 immunoreactivity and clinicopathological parameters in 53 patients with EEA.

Parameter	Total (*n* = 53)	17β-HSD2		
		+ (*n* = 19)	− (*n* = 34)	*p*-Value
Grade 1 (G1)	22	11	11	
2 (G2)	20	7	13	
3 (G3)	11	1	10	0.0480
Stage				
I, II	43	19	24	
III, IV	10	0	10	0.0218
LVI				
No	37	15	22	
Yes	16	4	12	0.2704
MI				
None or less than half	31	14	17	
More than half	22	5	17	0.0884
Androgen receptor				
Positive	40	17	23	
Negative	13	2	11	0.0629
Estrogen receptor				
Positive	37	15	22	
Negative	16	4	12	0.2704
Progesterone receptor				
Positive	33	17	16	
Negative	20	2	18	0.0023
Ki-67 LI median (min-max) (%)		20.4 (3–90)	30.6 (0–96)	0.0212

MI, myometrial invasion; LVI, lymphovascular invasion. We performed lymphadenectomy for 42 cases, and did not resect any lymph node in 11 cases (not resected).

## Supplementary Materials

The following are available online at http://www.mdpi.com/1422-0067/19/4/1139/s1.
