# Supplementary material for: 17β-Hydroxysteroid Dehydrogenase Type 2 Expression Is Induced by Androgen Signaling in Endometrial Cancer
Source: Int J Mol Sci. 2018 Apr 10;19(4):1139. doi: 10.3390/ijms19041139 (PMC5979403; doi:10.3390/ijms19041139)
Supplement: Supplementary file 1 [file ijms-19-01139-s001.pdf]

Supplemental Table 1. Clinicopathological characteristics of 19 patients whose hormonal concentrations were measured by LC-MS/MS (2012-2013).

Parameter	n (%)	Median (range)
Age		63.2 (51-76)
BMI		24.0 (17.1-31.0)
Grade 1 (G1)	7 (36.8)	
2 (G2)	6 (31.6)	
3 (G3)	6 (31.6)	
Stage Ⅰ	13 (68.5)	
Ⅱ	2 (10.5)	
Ⅲ	2 (10.5)	
Ⅳ	2 (10.5)	
MI		
No or less than half	10 (52.6)	
More than half	9 (47.4)	
LVI		
No	7 (36.8)	
Yes	12 (63.2)	
LN metastasis		
No	12 (63.2)	
Yes	0 (0)	
Not resected	7 (36.8)	
Adjuvant therapy		
No	9 (47.4)	
Yes	10 (52.6)	

BMI, body mass index; MI, myometrial invasion; LVI, lymphovascular invasion; LN, lymph node. We performed lymphadenectomy for 12 cases, and did not resect any lymph node in 7 cases (not resected).

Supplemental Table 2. Clinicopathological characteristics of 53 patients with EEA (1993-2001).

Parameter	n (%)	Median (range)
Age		61 (51-85)
Follow-up period (months)		85 (8-123)
Relapse or progression	7 (13.2)	
Death		
Total	6 (11.3)	
Death with EEA	5 (9.4)	
Death of the other cause	1 (1.9)	
Stage (FIGO 2009)		
I	45 (84.9)	
II	0 (0.0)	
III	6 (11.3)	
IV	2 (3.8)	
Grade		
G1	22 (41.5)	
G2	20 (37.7)	
G3	11 (20.8)	
MI		
None or less than half	31 (58.5)	
More than half	22 (41.5)	
LVI		
No	37 (69.8)	
Yes	16 (30.2)	
LN metastases		
Negative	39 (73.6)	
Positive	3 (5.7)	
Not resected	11 (20.7)	
Adjuvant therapy		
No	22 (41.5)	
Yes	31 (58.5)	

MI, myometrial invasion; LVI, lymphovascular invasion; LN, lymph node. We performed lymphadenectomy for 42 cases, and did not resect any lymph node in 11 cases (not resected).
